# Adaptation to cinnamaldehyde shapes *Pseudomonas aeruginosa* resistance to major antibiotics

**DOI:** 10.1128/jb.00180-25

**Published:** 2025-09-26

**Authors:** Eline Dubois, Susie Gaillot, Benoît Valot, Maxime Bour, Jean-Michel Brunel, Lison Schmidlin, Patrick Plésiat, Catherine Llanes

**Affiliations:** 1 Université Marie et Louis Pasteur, CNRS, Chrono-environnement (UMR 6249) 27000, Besançon, France; 2 Plateforme de Bioinformatique et Big data au Service de la Santé, UFR Santé, Université Marie et Louis Pasteur 27000, Besançon, France; 3 Centre National de Référence de la Résistance aux Antibiotiques, Laboratoire de Bactériologie, Centre Hospitalier Universitaire Jean Minjoz 55049, Besançon, France; 4 UMR_MD1, U-1261, Aix Marseille Université, INSERM, SSA, MCT 128791, Marseille, France; Geisel School of Medicine at Dartmouth, Hanover, New Hampshire, USA

**Keywords:** cinnamaldehyde, *Pseudomonas aeruginosa*, antibiotic resistance, efflux pumps, ATP synthase

## Abstract

In France, the use of essential oils to treat bacterial infections is common, with approximately 40% of cystic fibrosis patients regularly using these natural products to control infections caused by *Pseudomonas aeruginosa* often in combination with their antibiotic treatments. Previous research has demonstrated that prolonged exposure of *P. aeruginosa* PA14 to cinnamaldehyde (CNA), the main component of cinnamon essential oil, can select for mutants resistant to β-lactams through overproduction of the MexAB-OprM efflux pump, some of which are also hypersusceptible to aminoglycosides and colistin. We showed here that this hypersusceptibility is not due to an efflux defect, as the deletion of MexXY(OprM)—the specific efflux pump for aminoglycosides—still results in decreased minimum inhibitory concentrations of aminoglycosides. Genome sequencing of hypersusceptible mutants revealed mutations in the ATP synthase operon or its promoter (*atpIBEFHAGDC*). Surprisingly, although mutations in the *atp* operon reduced bacterial growth and ATP production, they are not uncommon in clinical strains. We found that ATP synthase alterations modified the respiratory chain and led to inner membrane hyperpolarization, likely enhancing positively charged antibiotic (aminoglycosides and colistin) uptake and susceptibility to these molecules. In addition, the modified respiratory chain increased the proton motive force, allowing the overproduction of the MexAB-OpM efflux pump, which protects bacteria from CNA and from the clinically relevant β-lactam antibiotics. Altogether, these results indicate a trade-off between CNA resistance and aminoglycoside/colistin susceptibility, a reaction that may question the survival of *P. aeruginosa* in the lung of CF patients possibly submitted to these therapeutic molecules.

**IMPORTANCE:**

In France, essential oils are widely used by cystic fibrosis patients (40%), often alongside antibiotic therapies, to help control *Pseudomonas aeruginosa* infections. Cinnamaldehyde from cinnamon essential oil appears to select for *P. aeruginosa* mutants that are resistant to β-lactam antibiotics due to the overproduction of the MexAB-OprM efflux pump and hypersusceptible to aminoglycosides and colistin. This increased susceptibility is associated with mutations in ATP synthase, which elevate the proton motive force (PMF) and facilitate both (i) increased uptake of positively charged antibiotics (aminoglycosides, colistin) and (ii) more efficient efflux of β-lactams via MexAB-OpM. Thus, the use of cinnamaldehyde may drive a trade-off in *P. aeruginosa* between β-lactam resistance and aminoglycosides/polymyxins susceptibility, potentially compromising bacterial persistence in the lung of patients.

## INTRODUCTION

Due to the increase in antibiotic resistance, many research groups have focused on studying the antimicrobial properties of plants and their extracts (1). It has been reported that oxygenated terpenoids found in essential oils (EOs), such as alcohols, aldehydes, esters, ketones, peroxides, and phenols, are responsible for strong antimicrobial activity and commonly used in self-medication to treat mild infections (2). In addition, a growing number of studies suggest that EOs could be useful as adjunctive therapy in cystic fibrosis patients chronically infected with *P. aeruginosa*, especially as their activity is independent of the resistance profile of the strains (3). The search for natural products to treat such infections has revealed that cinnamon EO, or its main component (70–80%), cinnamaldehyde (CNA), has significant bactericidal activity against *P. aeruginosa* with a minimum inhibitory concentration (MIC) lower than 800 µg/mL (4, 5). This activity is higher than that of other EOs, such as clove, geranium, lemon, orange, and rosemary (MICs ranging from 1,600 to over 12,800 µg/mL) (6).

However, if CNA is to be used clinically, it is important to be aware of possible resistance pathways in *P. aeruginosa* strains. Indeed, shortly after initial exposure to CNA and before the establishment of metabolic pathways to degrade this compound, *P. aeruginosa* exhibits a transient burst of efflux activity (from *t*_15 min_ to *t*_60 min_ after exposure) (7). This phenomenon involves at least four multidrug efflux systems of the RND family: MexAB-OprM, MexCD-OprJ, MexEF-OprN, and MexXY(OprM). Gene deletion experiments demonstrated that only the induction of MexAB-OprM limits CNA-promoted killing of *P. aeruginosa* during early exposure (7). This induction is caused by the activation of the NalC pathway, where a small protein, called ArmR, binds and sequesters MexR, the local repressor of *mexAB-oprM* (8). This leads to *mexAB-oprM* overexpression and subsequent higher resistance to CNA and antibiotic substrates (e.g., β-lactams and fluoroquinolones). However, the increase in MICs (four to eightfold) is transient and returns to basal levels once the CNA is metabolized (7). As the overproduction of MexAB-OprM is currently occurring in the clinical context (9), cross-resistance to CNA should not be rare and is important to study.

Until now, very few studies succeeded to obtain stable resistant mutants selected by EOs *in vitro*. In a previous work, we exposed the reference *P. aeruginosa* strain PA14 to toxic concentrations of CNA *in vitro*. After 10 days of exposure, we selected 10 CNA-resistant mutants (MIC = 900–1,100 µg/mL) with antibiotic cross-resistance (10). Most of them (8/10) overproduced MexAB-OprM pump after mutations in *nalC*. Interestingly, three of these 10 mutants were hypersusceptible to aminoglycosides and colistin and highly resistant to β-lactams for an unknown reason (10).

Aminoglycosides are effective against wild-type strains of *P. aeruginosa* despite its intrinsic low-level resistance to this class of antibiotics due to the production of the MexXY(OprM) efflux pump (11). The entry of aminoglycosides into Gram-negative bacteria is a multi-step process (12). First, these antibiotics interact with the bacterial outer membrane by electrostatic attraction of negatively charged lipopolysaccharides and phospholipids. This binding results in the displacement of divalent cations and an increase in membrane permeability, allowing access to the periplasmic space. A small fraction of aminoglycosides crosses the inner membrane using the motive force of protons in an energy-dependent manner. In the cytoplasm, aminoglycosides bind the 16S rRNA of the 30S ribosomal subunit, where they inhibit translation initiation, block translation elongation, and induce error-prone translation (13). Mistranslated proteins are hypothesized to damage the inner membrane, making it easier for the antibiotics to enter the cytoplasm. Consequently, the uptake of aminoglycosides can be inhibited by blocking electron transport and oxidative phosphorylation (14), and their activity is reduced under anaerobic conditions (15). Aminoglycoside hypersusceptible mutants have already been obtained *in vitro* following (i) a decrease in efflux due to alteration in the specific efflux pump MexXY(OprM) (16) or (ii) an increase in uptake across membranes (17).

In France, the use of EOs to treat bacterial infections is common. For instance, approximately 40% of cystic fibrosis patients regularly use these natural products often in combination with their antibiotic treatments (18). In this study, we analyzed the mechanisms by which two *P. aeruginosa* mutants selected by CNA became hypersusceptible to aminoglycosides and resistant to β-lactams in order to understand how iterative exposure to EOs can select for such an antibiotic resistance phenotype of this pathogen.

## RESULTS

### Efflux defect is not responsible for the hypersusceptibility to aminoglycosides in A1 and A3 mutants

Mutants A1 and A3 displayed hypersusceptibility to aminoglycosides, with MICs of tobramycin and gentamicin four to eightfold lower than those of their wild-type parent *P. aeruginosa* PA14. In addition, A1 had a reduced susceptibility to β-lactams (ticarcillin, aztreonam, and meropenem) and ciprofloxacin due to a mutation in *nalC* that led to the overexpression of the *mexAB-oprM* operon, as in the canonical *nalC* mutant A2 (*mexB*, Table 1). Therefore, in A1, tolerance to CNA is due to the activity of the MexAB-OprM pump, whereas in A3, the cause of tolerance to CNA is unclear (10).

**TABLE 1:** Features of the CNA-resistant mutants of *P. aeruginosa* hypersusceptible to aminoglycosides and colistin^*e*^

Strains/mutants^*a*^	Sequence			Efflux expression^*c*^		MICs (µg/mL) of antibiotics and CNA^*d*^								
	nalC	Efflux	atpD/atpI	mexB	mexY	TIC	ATM	MEM	IPM	TMN	GEN	CIP	CST	CNA
Reference strain														
PA14	WT	WT	WT	1	1	32	4	0.5	1	0.25	0.5	0.125	1	700
PA14 + CNA^*b*^	WT	WT	WT	3.2	5.5	64	16	2	1	1	2	1	2	NR
Spontaneous CNA^R^ mutants														
A1	−A_486_	AB^+^	−C_−95_(atpI)	10.3	0.9	256	32	1	1	0.03	0.125	0.5	0.5	900
A2	T_24_P	AB^+^	WT	2.5	0.8	128	16	1	1	0.25	0.5	0.25	1	900
A3	WT	WT	P_305_S (AtpD)	1.3	1.3	64	8	0.5	1	0.03	0.125	0.125	0.25	800
Constructed mutants														
PA14ΔXY	WT	∆XY	WT	ND	ND	32	4	0.5	1	0.125	0.125	0.125	1	700
A1∆XY	−A_486_	AB^+^∆XY	−C_−95_ (atpI)	ND	ND	256	32	1	1	0.015	0.03	0.5	0.5	900
A3∆XY	WT	∆XY	P_305_S (AtpD)	ND	ND	64	8	0.5	1	0.015	0.03	0.125	0.25	900

^*a*^ Mutants A1, A2, and A3 were obtained in a previous study (10). Mutants A2 and A3 harbor one mutation, and A1 harbors two mutations.

^*b*^ NalC derepression resulting from 30 min exposure to 512 μg/mL CNA. The results are published in reference 7.

^*c*^ Expressed as a ratio to wild-type reference strain PA14. Mean values were calculated from two-independent bacterial cultures each assayed in duplicate. The transcript levels of mexB > 2-fold, and mexY > 5-fold those of PA14 were considered as significantly increased because they were associated with a ≥2-fold higher resistance to respective pump substrates (19).

^*d*^ TIC, ticarcillin, ATM, aztreonam and MEM, meropenem are substrates of MexAB-OprM; IPM, imipenem is not a substrate of efflux; TMN, tobramycin and GEN, gentamicin are substrates of MexXY(OprM); CIP, ciprofloxacin is a substrate of two pumps [MexAB-OprM and MexXY(OprM)]; CST, colistin is not a substrate of efflux in wild-type strains; CNA, cinnamaldehyde. ND: not determined; NR non-relevant.

^*e*^ Status of *atp* cluster (wild-type or mutated) is gray shaded.

Since aminoglycosides are specific substrates of MexXY(OprM), we investigated whether this pump was involved in the hypersusceptibility of the A1 and A3 mutants. RT-qPCR showed that *mexY* in mutants A1 and A3 had the same basal expression as in the parental strain PA14 (Table 1). To determine whether the MexXY(OprM) efflux pump functions normally, we deleted *mexXY* by homologous recombination. Deletion of *mexXY* in the A1 and A3 mutants (producing mutants named A1∆XY and A3∆XY, respectively) reduced the MICs of aminoglycosides (up to fourfold) as observed in the *mexXY*-deleted PA14 mutant PA14∆XY (Table 1). A comparable result was obtained when the strains were treated with the protonophore CCCP, which prevents the activity of RND efflux systems (20), especially for the better substrate of MexXY(OprM), gentamicin (21) (Table S1). This demonstrates that MexXY(OprM) remained active in these hypersusceptible mutants and that the enhanced activity of aminoglycosides was mediated by another mechanism.

### Mutants hypersusceptible to aminoglycosides have altered ATP synthase function

In order to discover the mechanism involved in the hypersusceptibility to aminoglycosides, we compared the genomic sequences of the A1 and A3 mutants with that of PA14. It revealed that the *atp* cluster was mutated in hypersusceptible mutants A1 and A3 (Table 1). We have previously found that mutant A3 had a substitution in the *atpD* gene, encoding the β chain of ATP synthase, which forms the rotor allowing ATP production from ADP and phosphate (Fig. S1) (10). This mutation results in a P_305_S substitution that is expected to affect the ATP synthase function according to the PolyPhen-2 threshold (score > 0.85) (http://genetics.bwh.harvard.edu/pph2/). The A1 mutant was known to have only a *nalC* mutation (10), but genome sequencing revealed an additional previously unidentified mutation upstream of the *atpI* gene (–C, 95 bp upstream of the ATG of *atpI*). The impact of the –C deletion upstream of *atpI* remains uncertain, as this region has not yet been characterized. *In silico* analysis of the putative promoter region of *atpI* (http://www.softberry.com/) indicated that the deletion is located between boxes –10 and −35, shortening the length of the spacer from 14 to 13 bp (Fig. S1).

**Fig 1:**
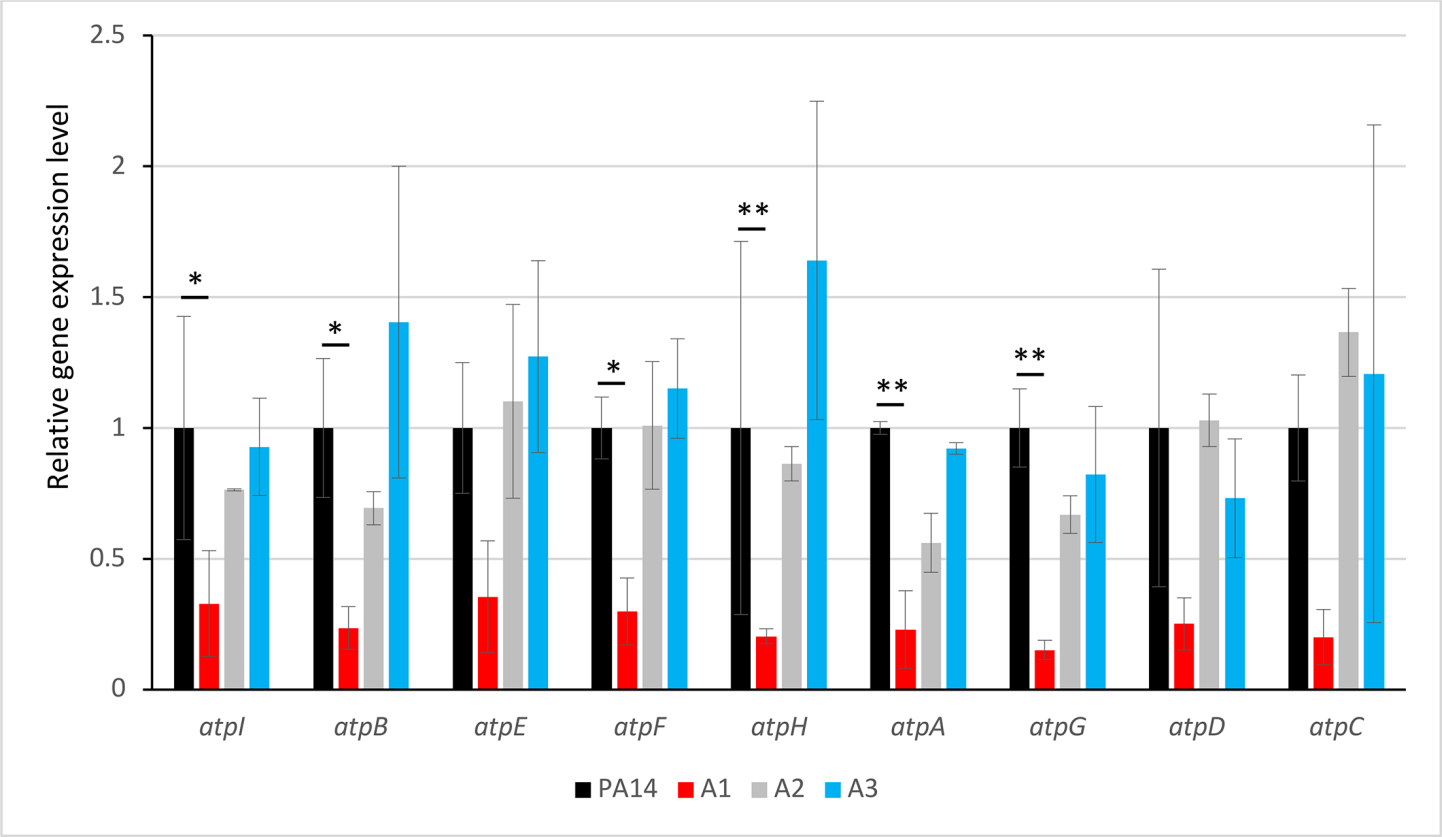
Analysis of the differential gene expression of the *atpI*, *atpB*, *atpE*, *atpF*, *atpH*, *atpA*, *atpG*, *atpD*, and *atpC* genes measured by RT-qPCR. Differences in cycle threshold (∆Ct) values corrected by the efficiencies of each gene were calculated according to reference 23. Mutants A1 and A3 were compared to the parental strain PA14. The A2 mutant was used as a control resistant to CNA but without alteration in the *atp* cluster. Significance was determined by one-way ANOVA, followed by Dunnett’s test (*: *P-value* < 0.05; **: *P-value* < 0.01).

To determine whether this promoter modification affects transcription, we measured the expression level of all the genes in the *atp* cluster (*atpI*, *atpB*, *atpE*, *atpF*, *atpH*, *atpA*, *atpG*, *atpD*, and *atpC*) by RT-qPCR (Fig. 1). The results show that A1 exhibits reduced expression levels for all genes in the *atp* cluster compared with PA14, A2, and A3, suggesting (i) that the mutation upstream of *atpI* prevents correct binding of RNA polymerase to the promoter, and (ii) that these genes function as an operon, as already shown in *E. coli* (22). Interestingly, the sole inactivation of the *atpI* gene by the MrT7 transposon (from the PA14 mutant library, University of Washington) increased the susceptibility to aminoglycosides, as shown by the PA14*atpI*::MrT7 antibiogram (Fig. S2).

### Altered ATP synthase correlates with reduced fitness and impaired ATP production

**Fig 2:**
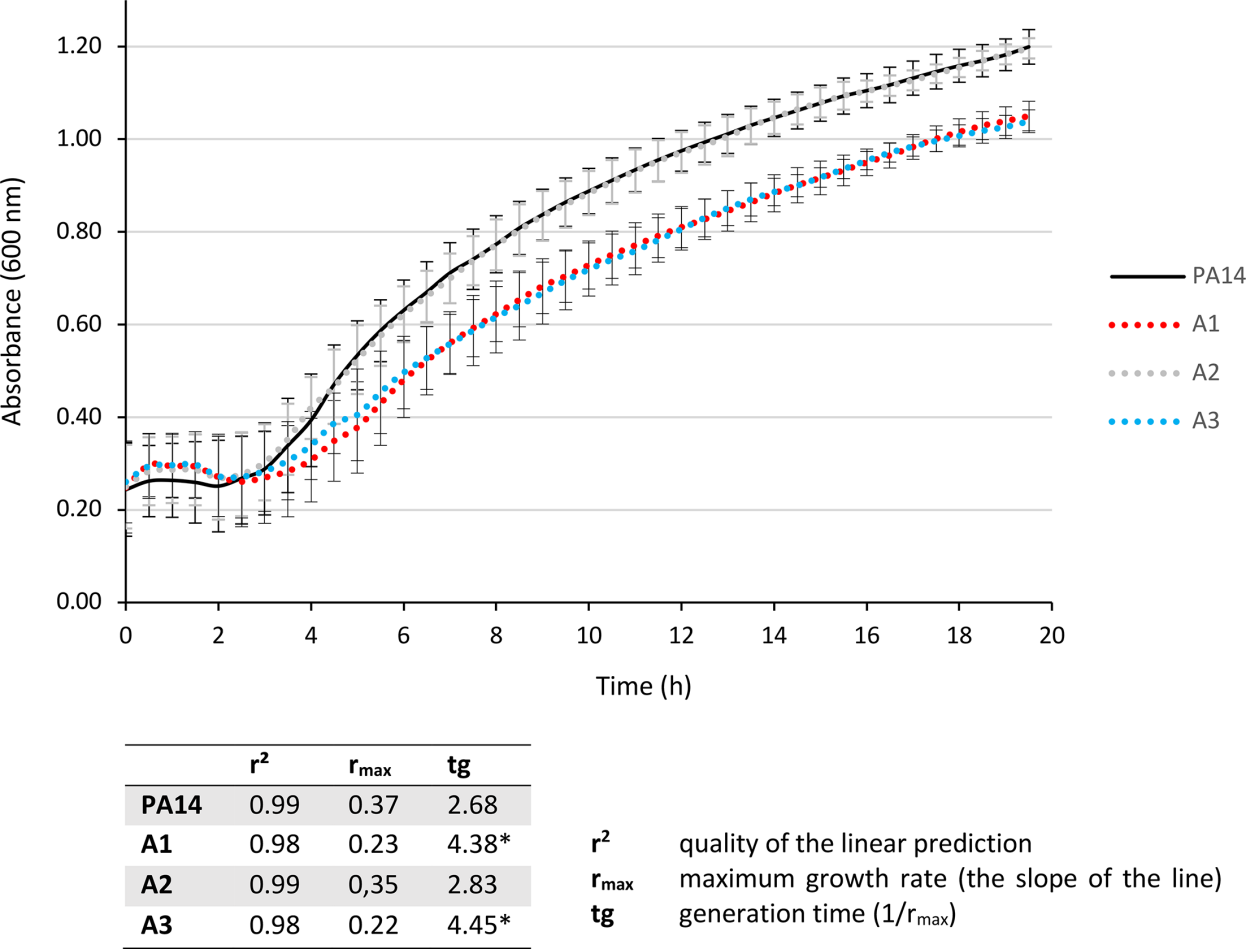
Growth curves for the A1 and A3 mutants affected in ATP production. PA14 and A2 were used as control. The generation time (tg) was calculated from the growth rate (see table). Significance was determined on generation time by one-way ANOVA, followed by Dunnett’s test: A1 and A3 are significantly different from PA14 (*: *P-value* < 1.10^−6^).

Given that A1 and A3 mutants are affected in the ATP synthase function, we hypothesized that these mutants have reduced fitness and impaired growth. Bacterial growth kinetics over 18 h confirmed that A1 and A3 had an increased generation time (×1.6) compared to the PA14 parental strain or the canonical *nalC* mutant A2 (Fig. 2). To determine the impact of ATP synthase alteration in these mutants, we measured ATP levels in lysed cells by luminescence. Since preliminary kinetic assays on the reference strain PA14 showed that ATP production was optimal during the exponential growth phase (data not shown), we, therefore, took measurements at the beginning (A_600 nm_ = 0.3) of the exponential phase. As expected, ATP production was lower than that of PA14 for all mutants (Fig. S3), including A2, which was unaltered in ATP synthase but overproduced MexAB-OprM in exchange for protons from the proton motive force (PMF). Hence, the decrease in the ATP synthase activity eliminates a key competitor for protons and facilitates cytoplasmic pH homeostasis in overproducers of RND efflux systems (24). By the way, the A2 *nalC* mutant (which has a wild-type *atp* operon) showed slightly reduced expression of the *atp* genes (Fig. 1), although this was not significant.

### Cell surface charge remains unchanged in ATP synthase mutants

As efflux cannot fully explain hypersusceptibility to aminoglycosides, we investigated whether these antibiotics could better penetrate inside bacteria through membranes. Aminoglycosides are polycationic molecules at neutral pH, and the electrostatic interaction of their amine group with the phosphate residues of lipopolysaccharides is believed to promote their penetration through the outer membrane. Consequently, changes in negatively charged cell surface charges have been previously correlated with modification in susceptibility to these antibiotics (25). We assessed the bacterial surface charges through the measurement of the Zeta potential and found no difference between strain PA14 (−35.10 ± 5.59 mV) and derivative mutants A1 (−37.52 ± 5.26 mV), A2 (−35.35 ± 5.02 mV), and A3 (−34.35 mV ± 6.13).

### Alteration in ATP synthase function correlates with minor modifications in respiratory chain activity

**Fig 3:**
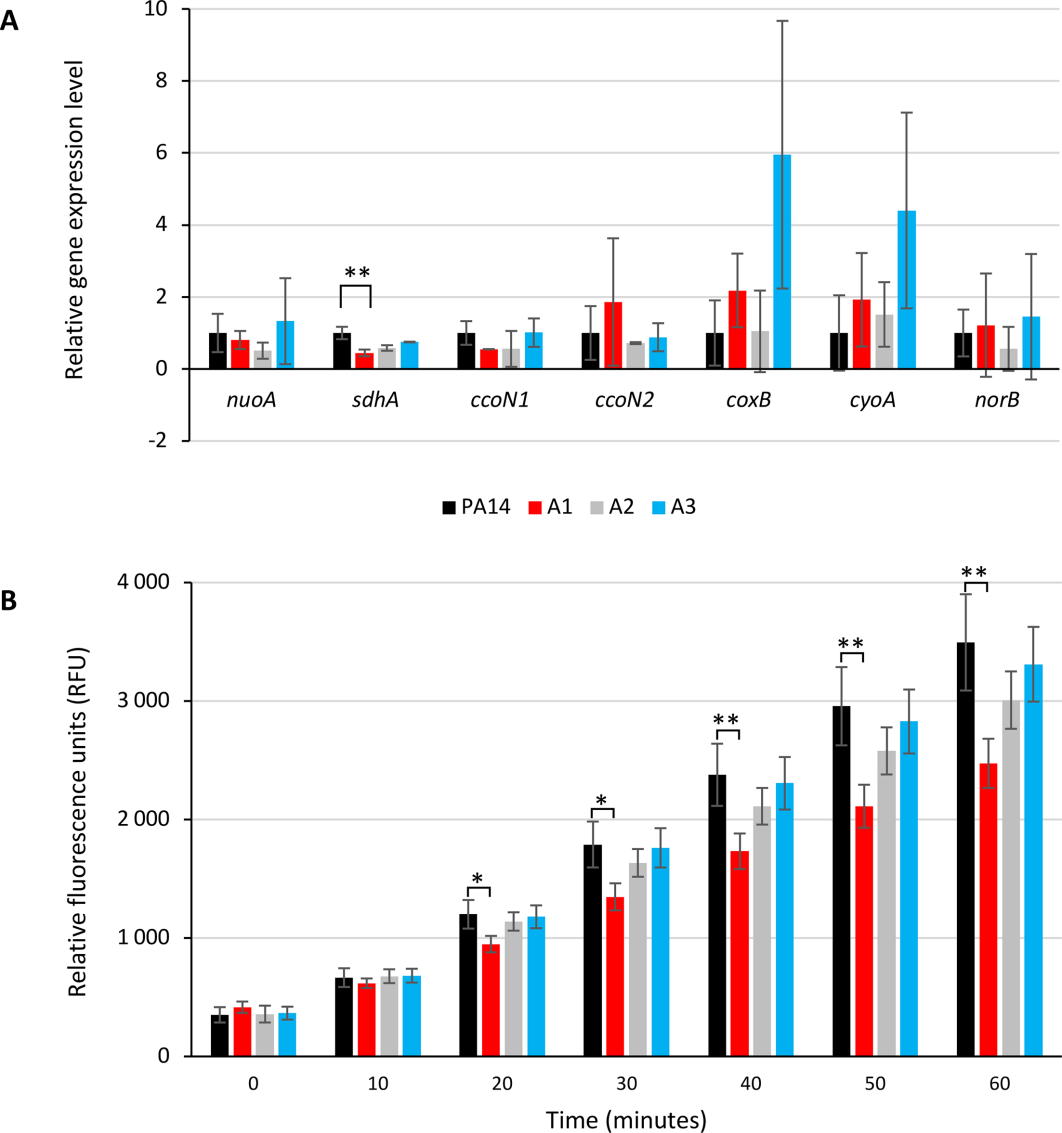
Respiratory activity of the *P. aeruginosa* hypersusceptible mutants. (**A**) Differences of the cycle threshold (∆Ct) values relative to the wild-type PA14 strain of the selected genes encoding peptides of transporters of the respiratory chain *nuoA* (NADH oxidoreductase from complex I), *sdhA* (succinate dehydrogenase from complex II), and terminal oxidases: *ccoN1* (constitutive cytochrome c oxidase ccb_3_1)*, ccoN2* (cytochrome c oxidase ccb_3_2 inducible in anaerobiosis)*, coxB* (cytochrome c oxidase aa_3_ inducible by nutriment starvation)*, cyoA* (quinol oxidase bo_3_ inducible by iron starvation)*,* and *norB* (NO reductase, inducible in anaerobiosis). Significance was determined by one-way ANOVA, followed by Dunnett’s test. (**B**) Comparison of the respiration status over time. The metabolic activity measured at the end of the exponential phase (A_600nm_= 1) is determined based on the reduction of the non-fluorescent product resazurin to the fluorescent resorufin by the dehydrogenases present in cells. Significance was determined by one-way ANOVA, followed by Dunnett’s test (*: *P-value* < 0.05; **: *P-value* < 0.01).

If greater uptake of aminoglycosides is not associated with the outer membrane, it may be related to the energy provided by the electrochemical potential of the cytoplasmic membrane and the flow of electrons through the membrane respiratory chain (12). Indeed, ATP synthase functions in conjunction with the PMF, and any alterations to this enzyme are likely to impact proton translocation across the inner membrane. Given that the processes of proton consumption and generation are precisely balanced, disturbances in ATP synthase can have feedback effects on the respiratory chain. Bacteria use mostly NDH-1 enzymes and terminal respiratory oxidases to pump protons through the cytoplasmic membrane (26). *P. aeruginosa* possesses five terminal oxidases for aerobic respiration (27): three cytochrome *c* oxidases (Cbb3-1, Cbb3-2, and Aa3) and two quinol oxidases (Bo3 and Cio). These terminal oxidases are expected to have specific affinities for oxygen and varying efficiencies in proton translocation (Table S2). Additionally, the respiratory chain is also branched with the denitrification enzymes that reduce nitrogen oxides (e.g., NO reductase) enabling *P. aeruginosa* to grow under anaerobic conditions in the presence of nitrate or nitrite (27). We, therefore, compared the expression levels of selected respiratory genes in strains A1, A2, and A3 (Fig. S4) with those of the parental strain PA14. The results showed that of all the genes tested, only *sdhA* was significantly downregulated in the A1 mutant (Fig. 3A). This gene encodes the succinate dehydrogenase, an enzyme common to both the tricarboxylic acid (TCA) and the respiratory chain, not involved in proton pumping. Consistent with this result, the measurement of NADH oxidation by using resazurin revealed that only mutant A1 was affected in respiratory activity (Fig. 3B). Moreover, in agreement with the similar expression of *norB* (which encodes the cytochrome b subunit of NO reductase activated under anaerobic conditions), the A1 and A3 mutants showed no difference in growth between aerobic and anaerobic conditions (data not shown).

### Alteration in ATP synthase function correlates with proton gradient modifications beneficial to MexAB-OprM

Finally, since none of the proton-pumping sites in the respiratory chain were significantly altered, we hypothesized that the reduction in ATP synthase activity and the subsequent return of protons to the cytoplasm increased the proton pool and the PMF of mutants A1 and A3. To demonstrate this, we compared the fluorescence of BCECF/AM [2′,7′-bis-(2-carboxyethyl)-5-(and-6)-carboxyfluorescein/acetoxymethyl ester], a molecular marker whose fluorescence increases with rising cytoplasmic pH, in A1, A2, A3, and PA14. Mutants A1 and A3 showed higher fluorescence (Fig. S5) than PA14, indicating a higher cytoplasmic pH and indirectly a lower periplasmic pH due to the accumulation of protons in this space. This effect is further enhanced by the use of DCCD (N,N′-dicyclohexylcarbodiimide), an ATP synthase inhibitor that blocks protons in the periplasmic space (Fig. S5); except in the A3 mutant where DCCD has no effect, confirming that ATP synthase is inactivated by the P_305_S mutation in *atpD*. An increased PMF could also provide additional protons available for the MexAB-OprM efflux. This hypothesis is supported by the elevated MICs of antibiotics that are substrates of this pump (e.g., ticarcillin, aztreonam, and ciprofloxacin) in the A1 mutant compared to the canonical *nalC* A2 mutant (Table 1). Incidentally, MexAB-OprM, which is expressed at basal levels in A3 (in the absence of a mutation in *nalC*), could have a higher activity compared to the PA14 strain due to the alteration in the ATP synthase, as indicated by the twofold increase in the MIC of ticarcillin and aztreonam (Table 1). Additionally, the MICs of ticarcillin and aztreonam were further increased by twofold upon treatment with DCCD in strains with basal expression of *mexAB-oprM*, such as PA14 and A3 (Table S3).

### ATP synthase mutants display hyperpolarization of their inner membrane associated with better uptake of the charged antibiotics aminoglycosides and colistin

**Fig 4:**
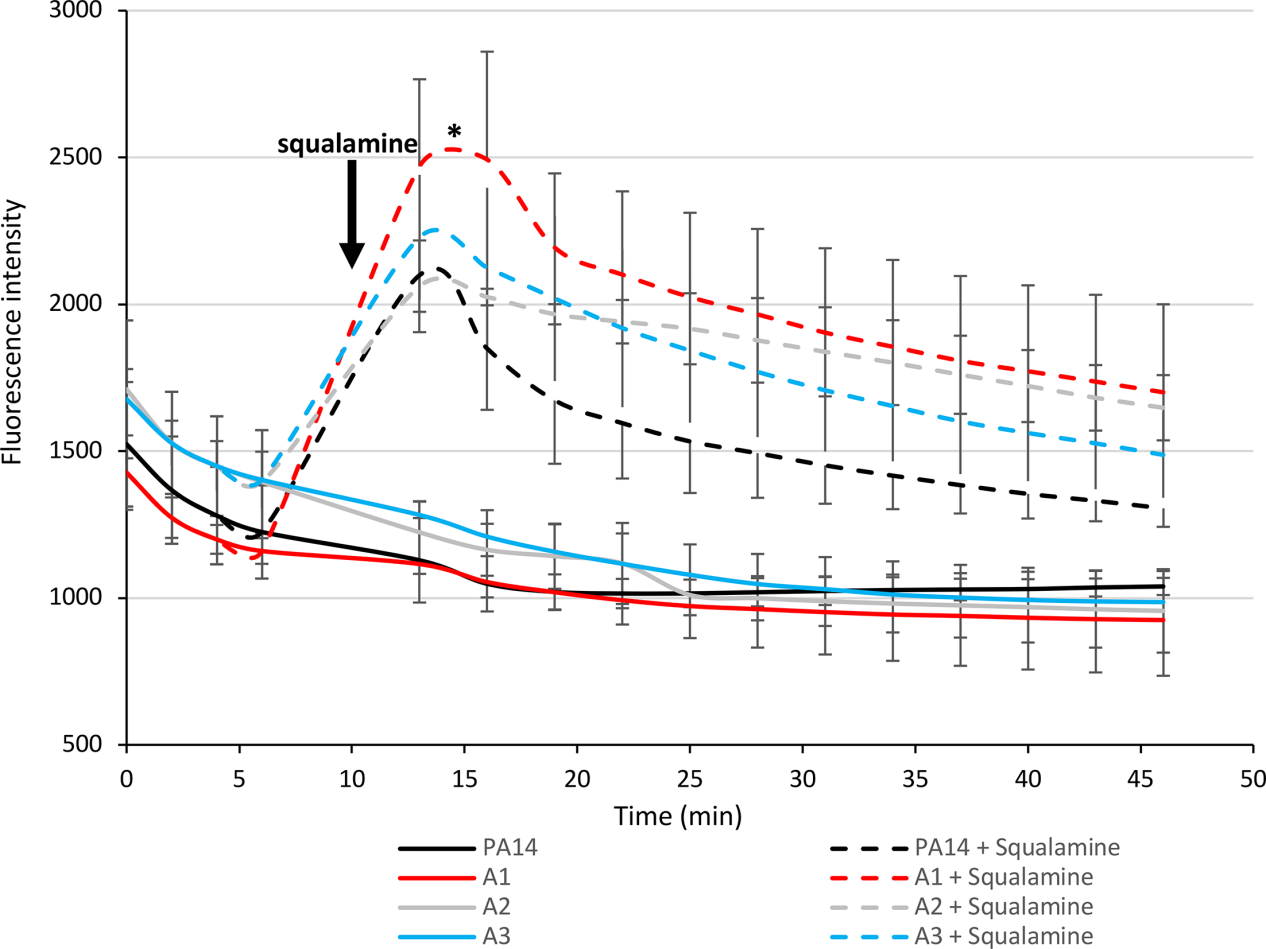
Measurement of the membrane potential (∆ψ) in hypersusceptible mutants A1 and A3. DiSC_3_ is a voltage-sensitive dye that accumulates on polarized membranes, leading to fluorescence quenching. Upon membrane depolarization by squalamine (at *t* = 10 min), the dye is released into its surrounding environment, which is detected as a change in fluorescence intensity (*λ*_ex_ = 622 nm and *λ*_em_ = 670 nm). PA14 and A2 were included as controls. Statistical significance was assessed 15 min after squalamine injection using one-way ANOVA, followed by Dunnett’s post-hoc test. The fluorescence intensity of strain A1 was significantly higher than that of PA14 (*: *P-value* < 0.05).

The proton gradient resulting from the balance between proton translocation by the respiratory chain and proton consumption by ATP synthase (and other proton-consuming mechanisms, such as efflux and flagellar mobility) generates both a chemical gradient (∆pH/∆[H^+^]) and an electric membrane potential (∆ψ). It has long been known that the magnitude of the membrane potential significantly affects the translocation of aminoglycosides (28) and antimicrobial peptides (29). We, therefore, hypothesized that, conversely, *atp* mutants might be more susceptible to aminoglycosides and possibly to colistin as well due to hyperpolarization of their membrane and an increase in ∆ψ. To test this hypothesis, we assessed the membrane potential of the mutants and compared it to that of PA14 using the fluorescent dye DiSC3(5), which has increased affinity for hyperpolarized membranes. After the addition of the natural aminosterol squalamine, which disrupts the bacterial membrane and causes DiSC3(5) leakage, *atp* mutants A1 and A3 indeed exhibited greater fluorescence than PA14 or A2, indicating membrane hyperpolarization (Fig. 4); incidentally, their MIC of aminoglycosides decreased by at least a factor of 2 when squalamine was added to the medium (Table S4). This state likely enhances aminoglycoside translocation, leading to hypersusceptibility to these antibiotics. Consistent with this, the addition of the ATP synthase inhibitor DCCD induced a similar phenotype (Table S3).

### Clinical strains of *P. aeruginosa* carry mutations in ATP synthase cluster

To investigate whether mutations in the *atp* cluster or its promoter can be selected *in vivo*, the genomes of 2,500 clinical strains of *P. aeruginosa* were analyzed (data from the CNR). Mutations predicted to be deleterious according to PolyPhen-2 (http://genetics.bwh.harvard.edu/pph2/) were identified in 3.8% of isolates (Table S5), a third of which (32%) were isolated from patients with cystic fibrosis, proving that these mutations can be selected in clinical strains that are still pathogenic. However, the accumulation of aminoglycoside resistance mechanisms in these strains masked the effect of atp cluster mutations on proton translocation. This raises the question of the real benefit of these *atp* mutations *in vivo*.

## DISCUSSION

In a previous study (10), we showed that long-term exposure to CNA from cinnamon essential oil can select for mutations in efflux regulatory genes (*nalC*) in the reference strain PA14 and more readily in strains isolated from cystic fibrosis patients. In this work, we have shown that mutations in the *atp* operon are also selected for by CNA, although this may be detrimental to the bacteria by impairing their fitness. Some of these same genes (*atpF*, *atpC*, *atpH*, *atpG*, *atpD*, and *atpA*) were transiently downregulated during short-term treatment with CNA compared to other genes encoding membrane proteins (Table S6), suggesting that the advantage in resisting CNA is significant, probably due to proton conservation for efflux facilitating the export of toxic compounds from the bacterium.

As the first response to CNA is the overproduction of the efflux pump MexAB-OprM, it is legitimate to question the role of this mechanism in the overall cellular response. Previous studies have demonstrated the links between efflux, proton influx across the inner membrane, membrane hyperpolarization, and the redox state of the cell (30), suggesting that efflux could play a crucial role in maintaining this equilibrium. For example, high membrane potential and increased cellular *reactive oxygen species* (ROS) levels enhance the efflux of aminoglycosides and fluoroquinolones via the RND efflux pumps MexAB-OprM and MexXY(OprM), leading to increased antibiotic resistance (31). This mechanism could explain why MexAB-OprM appears abnormally efficient in the A1 mutant (two times more resistant to antibiotic substrates than the canonical *nalC* mutant A2) and in the A3 mutant, which harbors a wild-type *nalC* (Table 1).

However, the selection of ATP-defective mutants has side effects, making mutants A1 and A3 more susceptible to aminoglycosides and polymyxins. In *Staphylococcus aureus*, the ATP synthase alteration also sensitizes bacteria to polymyxins (32) and gentamicin (33). Similarly, the inhibition of ATP synthase by venturicidin A reduces gentamicin MICs by 2- to 16-fold against *P. aeruginosa* (34).

Consistent with our findings showing that ATP synthase deficiency affects membrane potential, previous studies on *Corynebacterium glutamicum* and *S. aureus* have also reported that ATP synthase-deficient mutants exhibit an increased membrane potential compared to wild-type strains (33, 35). This phenomenon is not restricted to bacteria; in human cells, the inhibition of ATP synthase by the ATPase inhibitory factor 1 (IF1) increases mitochondrial membrane potential and ROS production (36). The uptake of aminoglycosides across bacterial membranes is dependent on membrane potential (∆ψ), which affects their ability to reach their intracellular target and reduces their activity against various bacterial species. In *E. coli*, a decrease in ∆ψ from −149 to −129 mV results in a twofold increase in the MIC of the aminoglycoside dihydrostreptomycin (28). This phenomenon is also observed in the translocation of antimicrobial peptides, such as colistin (29). Besides, hypersusceptible mutants A1 and A3 exhibit a two and fourfold decrease in MIC to colistin, respectively, compared to PA14 (Table 1), and the indirect inhibition of ATP synthase by CCCP increases the susceptibility of PA14 to colistin (Table S1). However, the only definitive way to demonstrate enhanced uptake of positively charged antibiotics is to measure the intracellular concentrations of these compounds in the A1 and A3 mutants and compare them in the PA14 strain.

To conclude, this study highlights the complex relationship between dissipation of PMF by ATP synthase activity and by efflux mechanisms. Perturbation of this equilibrium leads to (i) increased aminoglycoside uptake, which is favored by a higher ∆ψ, and (ii) increased efflux of antibiotics, such as β-lactams, driven by an elevated proton gradient. ATP synthase inhibition is already used to combat bacterial pathogens, as illustrated by bedaquiline approved by the Food and Drug Administration in 2012 to treat *Mycobacterium tuberculosis*. Although it may enhance the efficacy of aminoglycosides and other cationic antimicrobial peptides in *P. aeruginosa*, it can reduce the activity of MexAB-OprM substrate β-lactams, thus compromising new applications for existing antibiotics. Further experiments are needed at the end of this work to identify whether such mutants could be selected *in vivo* after treatment with antibiotics or essential oils. These experiments should also determine how the mutants can persist in competition with non-mutated *atp* strains under various concentration regimens despite their decreased fitness.

## MATERIALS AND METHODS

### Bacterial strains, plasmids, and growth conditions

Three mutants (A1, A2, and A3) derived from the reference strain *P. aeruginosa* PA14 and obtained after long-term exposure to CNA (10) were used in this study (Table S7). All bacterial cultures were incubated at 37°C in Mueller-Hinton broth (MHB) with adjusted concentrations of Ca^2+^ (20 to 25 µg/mL) and Mg^2+^ (10 to 12.5 µg/mL) (Becton Dickinson and Company, Cockeysville, MD, USA) or on Mueller-Hinton agar (MHA) (Bio-Rad, Paris, France) supplemented with antibiotics as needed. Cinnamaldehyde (CNA) and dimethyl sulfoxide (DMSO) were obtained from Sigma-Aldrich (Saint-Quentin Fallavier, France).

### Bacterial kinetic assays

Bacterial suspensions adjusted to an A_600nm_ of 0.01 in MHB were cultured in microplates for 20 h at 37°C with agitation (110 rpm). The growth was monitored using a Spark spectrophotometer (Tecan, Männerdorf, Switzerland) equipped with a humidity cassette to prevent evaporation. The bacterial growth was measured every 30 min by recording the absorbance at A_600 nm_.

### Genome sequencing**,** accession numbers, and *in silico* analysis

Whole-genome sequencing (WGS) of *P. aeruginosa* mutants A1, A2, and A3 was performed with the Illumina technology using 2 × 150 paired-end reads (Microsynth Society, Balgach, Switzerland) as previously reported (10). The complete genomic sequences have been deposited in the National Center for Biotechnology Information database under BioProject accession number PRJNA663565. The PolyPhen-2 (http://genetics.bwh.harvard.edu/pph2/) algorithm was used to predict the effects of identified substitutions, while Softberry (http://www.softberry.com/) was utilized to locate promoter regions.

### Drug susceptibility testing

The minimum inhibitory concentrations (MICs) of antibiotics were determined in triplicate using the standard microdilution method with Sensititre Gram-negative plates (Thermo Fisher, Illkirch-Graffenstaten, France) and interpreted according to the guidelines of the European Committee on Antimicrobial Susceptibility Testing (EUCAST [http://www.eucast.org]). The MIC values of CNA were obtained using a homemade microdilution method with MHB as the growth medium and 1% DMSO as the solvent. Antibiograms were performed three times using the agar diffusion technique following the recommendations of the “Comité de l’Antibiogramme de la Société Française de Microbiologie” (CA-SFM, 2024). A 0.5 McFarland bacterial suspension (approximately 10^8^ CFU/mL) was streaked onto MHA plates. Antibiotic discs (BioRad, Marnes-la-Coquette, France) were placed on the seeded plate. After 18 h of incubation at 37°C, the inhibition diameters were measured.

### Gene transcript quantification by RT-qPCR

Specific gene expression levels were quantified using real-time quantitative PCR (RT-qPCR) following reverse transcription, as described previously (37). In brief, 2 µg of total RNA was reverse-transcribed using the ImpromII reverse transcriptase according to the manufacturer’s instructions (Promega, Madison, WI). The quantification of specific cDNA was performed using a Rotor-Gene RG6000 real-time PCR instrument (Qiagen, Courtaboeuf, France) with the QuantiTect SYBR Green PCR Kit (Qiagen). Primers were designed based on sequences from the *Pseudomonas* Genome Database version 2 (Table S8), with *rpsL* transcripts serving as internal controls. The mRNA levels of target genes were normalized to the *rpsL* levels for each strain and expressed as differences in cycle threshold (Ct) values corrected for efficiencies relative to the wild-type PA14 strain used as the reference. Mean gene expression values were calculated from three independent bacterial cultures, each assayed in duplicate.

### Construction of efflux-defective mutants

MexXY-defective derivatives were constructed from PA14, A1, and A3 mutants using the suicide plasmid pKNG101 (Table S7). The recombinant plasmid was generated via assembly cloning, utilizing the NEBuilder Hi-Fi DNA Assembly Cloning Kit (New England Biolabs, France), with the deleted chromosomal region amplified using appropriate primers (Table S8). The assembly products were directly used to transform competent *Escherichia coli* strain CC118*λpir* (Table S7). The recombinant plasmid containing the desired inserts was then transferred to *P. aeruginosa* PA14, A1, or A3 by conjugation. Following homologous recombination, transconjugants were selected on *Pseudomonas* isolation agar (PIA; Becton Dickinson) containing 2,000 µg/mL streptomycin. Excision of the undesired pKNG101 sequence was achieved by plating transformants on M9 plates (8.54 mM NaCl, 25.18 mM NaH_2_PO_4_, 18.68 mM NH_4_Cl, 22 mM KH_2_PO_4_, 2 mM MgSO_4_, 0.8% agar, pH 7.4) containing 5% (wt/vol) sucrose. Negative selection on streptomycin-containing MHA was used to identify transconjugants that had lost the plasmid. Allelic exchange was verified by PCR, confirming the deletion of a 4,329 bp region in the *mexXY* genes.

### Measurement of ATP production by luciferase bioassay

Luminescence due to luciferin oxidation by luciferase was recorded from the PA14 strain and its derivative mutants during exponential growth in MHB. Total ATP levels were measured from lysed cells using the Biofax A Kit (Yelen Analytics, Marseille, France) according to the manufacturer’s instructions. For this purpose, an overnight culture of strain PA14 or its mutants was diluted to an A_600 nm_ of 0.1 in 15 mL of fresh MHB and incubated at 37°C with agitation (250 rpm). When the cultures reached an A_600 nm_ of 0.3, luminescence was recorded using a Synergy H1 microplate reader (Biotek Instruments, Winooski, USA). Data are presented for three independent cultures.

### Measurement of respiratory status

Bacterial respiratory rates were quantified using alamarBlue dye (Thermo Fisher Scientific), the active component of which is resazurin. Bacteria were grown to an OD_600 nm_ of 1.0. One hundred and eighty microliters of bacterial culture was mixed with 20 µL alamarBlue dye and added to one well of a black 96-well plate. Fluorescence was monitored every 10 min for 1 h (λ _excitation_= 560 nm, λ _emission_= 590 nm) using a Spark plate reader (Tecan, Männedorf, Switzerland). Data represent the mean ± standard deviation of results from three biological replicates.

### Measurement of proton motive force (PMF)

To quantify the proton motive force of the strains studied, liquid cultures were calibrated at OD_600 nm_=0.1 in MHB and incubated at 37°C with agitation. When the absorbance reached OD_600 nm_=0.8, 1 mL of each solution was incubated with the ATP synthase inhibitor DCCD (50 µg/mL) for 20 min with stirring. These mixtures were washed twice with one volume of K_3_PO_4_ (pH 6, 50 mM), then once with one volume of KE buffer (K_3_PO_4_ pH6, 50 mM + EDTA 5 mM). After resuspension in 1 mL of KE buffer, 10 µL BCECF/AM (2 mM) was added, followed by incubation with agitation for 1 h. Bacterial cells were collected by centrifugation and resuspended in 120 µL KE buffer. After 4 h of incubation at 4°C, they were plated in a 96-well plate (2 µL/well + 200 µL KE buffer). Fluorescence (λ_excitation_ = 500 nm, λ_emission_ = 522 nm) was read using a Spark plate reader (Tecan, Männedorf, Switzerland). The protocol was adapted from reference 38. Data represent the mean ± standard deviation of results from three biological replicates.

### Measurement of membrane potential by fluorescence

Electric membrane potential (∆ψ) was measured by using 3,3′-diethylthiacarbocyanine iodide (DiSC3(5)), a voltage-sensitive dye that accumulates on polarized membranes, leading to fluorescence quenching. Bacteria were adjusted to an A_600 nm_ of 0.1 in MHB and grown until the A_600 nm_ reached 0.8. The cultures were then centrifuged at 3,000 *g* at 20°C, and the bacteria were washed twice with 1/5 vol of GHEPES buffer (5 mM HEPES, 5 mM glucose). The cells were subsequently resuspended in the same buffer to a concentration of 5.10^8^ CFU/mL. The fluorescent dye DiSC3(5) was added to a final concentration of 2 µM, allowing the dye to penetrate the bacterial membranes during 1 h of incubation at 37°C. The bacteria were then washed to remove any unbound dye, and fluorescence measurements (λ_excitation_ = 622 nm, λ_emission_ = 670 nm) were recorded for 10 min using a Spark plate reader (Tecan, Männedorf, Switzerland). At *t* = 10 min, squalamine (62.5 µM), a molecule known for its membrane depolarizing activity (39), was added, and fluorescence (corresponding to the release of the dye) was measured every 5 min during 45 min. Data represent the mean ± standard deviation of results from three biological replicates.

### Measurement of bacterial surface charge

The bacterial surface charge was assessed by measuring the Zeta potential. Mid-log phase cultures of strain PA14 and its derived mutants (approximately 10^7^ CFU/mL) were centrifuged at 5,200 *g* for 10 min. The pellets were resuspended in distilled water to a concentration of 4 McFarland units. Zeta potential was measured at 25°C using a folded capillary cell (DTS 1070; Malvern Instruments, Worcestershire, United Kingdom) in a Zetasizer Nano ZS (Malvern Instruments) equipped with a 633 nm HeNe laser and controlled by Zetasizer software v7.02. Bacteria were allowed to equilibrate for 120 s at 25°C before measurement. Zeta potential was determined from two independent bacterial cultures, each assayed in triplicate. The Smoluchowski equation was employed to calculate the potential from nine electrophoretic mobility determinations.

### Statistical analysis

Statistical analyses were carried out using R software. The conditions for applying ANOVA (normality of values and homoscedasticity) were verified using Bartlett’s and Shapiro’s tests. If the *P*-value at the end of the ANOVA was significant, then a Dunnett’s test was performed with PA14 as reference. Error bars represent standard deviations.

## Data Availability

The complete genomic sequences have been deposited in the National Center for Biotechnology Information database under BioProject accession number PRJNA663565.

## SUPPLEMENTAL MATERIAL

Fig. S1 to S5 and Tables S1 to S8 jb.00180-25-s0001.docx Fig. S1: Map of the cluster atpIBEFHAGDC encoding the subunits 445 of the ATP synthase of *P. aeruginosa*. Fig. S2: Antibiotics susceptibility profile of the transposition mutant PA14atpI::MrT7 inactivated in atpI compared with PA14. Fig. S3: Measurement of ATP production in A1, A2 and A3 mutants by luminescence. Fig. S4: Elements of *P. aeruginosa* respiratory chain. Fig. S5: Measurement of the Proton Motive Force (PMF) using fluorescence of BCECF.Table S1: Impact of CCCP (protonophore) on the MIC of aminoglycosides, β-lactams, and polymyxins antibiotics. Table S2: Different terminal oxidases of the *P. aeruginosa* respiratory chain. Table S3: Impact of DCCP (inhibitor of ATP synthase) on the MIC of aminoglycoside and β-436 lactam antibiotics. Table S4: Impact of squalamine on the MIC of aminoglycoside antibiotics. Table S5: Mutations in the atp cluster found in clinical strains of *P. aeruginosa* (Centre439 National de Reference de la resistance aux antibiotiques, Besançon, France). Table S6. Expression levels of genes in the atp cluster of reference strain PA14 at 15- and 30-min post-exposure to 350 μg/mL CNA (transcriptomic analysis from our laboratory data). Table S7. Strains and plasmids used in the study. Table S8. Primers used for gene inactivation, RT-qPCR, and sequencing experiments.

ASM does not own the copyrights to Supplemental Material that may be linked to, or accessed through, an article. The authors have granted ASM a non-exclusive, world-wide license to publish the Supplemental Material files. Please contact the corresponding author directly for reuse.
