# Supplementary material for: Adaptation to cinnamaldehyde shapes Pseudomonas aeruginosa resistance to major antibiotics
Source: J Bacteriol. 2025 Sep 26;207(10):e00180-25. doi: 10.1128/jb.00180-25 (PMC12548396; doi:10.1128/jb.00180-25)
Supplement: Fig. S1 to S5 and Tables S1 to S8 — Fig. S1: Map of the cluster atpIBEFHAGDC encoding the subunits 445 of the ATP synthase of P. aeruginosa. Fig. S2: Antibiotics susceptibility profile of the transposition mutant PA14atpI::MrT7 inactivated in atpI compared with PA14. Fig. S3: Measurement of ATP production in A1, A2 and A3 mutants by luminescence. Fig. S4: Elements of P. aeruginosa respiratory chain. Fig. S5: Measurement of the Proton Motive Force (PMF) using fluorescence of BCECF.Table S1: Impact of CCCP (protonophore) on the MIC of aminoglycosides, β-lactams, and polymyxins antibiotics. Table S2: Different terminal oxidases of the P. aeruginosa respiratory chain. Table S3: Impact of DCCP (inhibitor of ATP synthase) on the MIC of aminoglycoside and β-436 lactam antibiotics. Table S4: Impact of squalamine on the MIC of aminoglycoside antibiotics. Table S5: Mutations in the atp cluster found in clinical strains of P. aeruginosa (Centre439 National de Reference de la resistance aux antibiotiques, Besançon, France). Table S6. Expression levels of genes in the atp cluster of reference strain PA14 at 15- and 30-min post-exposure to 350 μg/mL CNA (transcriptomic analysis from our laboratory data). Table S7. Strains and plasmids used in the study. Table S8. Primers used for gene inactivation, RT-qPCR, and sequencing experiments. [file jb.00180-25-s0001.docx]

***Supplementary data***

**Table S1:** Impact of CCCP (protonophore) on the MIC of aminoglycosides, β-lactams and polymyxins antibiotics.

| **Strains** | **MICs of antibiotics (µg/mL)** | | | | | | | | | | | |
| --- | --- | --- | --- | --- | --- | --- | --- | --- | --- | --- | --- | --- |
|  | **Gentamicin** | | | **Tobramycin** | | | **Colistin** | | | **Ticarcillin** | | |
| ***+ CCCP (µg/mL)*** | ***0*** | ***10*** | ***50*** | ***0*** | ***10*** | ***50*** | ***0*** | ***10*** | ***50*** | ***0*** | ***10*** | ***50*** |
| **PA14** | 0.5 | 0.5 | 0.25 | 0.25 | 0.25 | 0.25 | 1 | 0.5 | 0.25 | 32 | 32 | 16 |
| **A1** | 0.125 | 0.06 | 0.03 | 0.06 | 0.03 | 0.03 | 0.5 | 0.25 | 0.25 | 256 | 128 | 128 |
| **A2** | 0.5 | 0.5 | 0.25 | 0.25 | 0.25 | 0.25 | 1 | 0.5 | 0.5 | 128 | 128 | 64 |
| **A3** | 0.125 | 0.06 | 0.03 | 0.06 | 0.06 | 0.015 | 0.25 | 0.25 | 0.25 | 64 | 64 | 32 |

CCCP is a protonophore disrupting the proton motive force (PMF) by transporting protons across the inner membrane; it is used to prevent RND efflux systems activity. Gentamicin and tobramycin are substrates of the MexXY(OprM), ticarcillin is a substrate of MexAB-OprM efflux system, and colistin is not an efflux substrate.

| **Terminal oxidases** | **Operon** | **O_2_ affinity** | **Conditions of production** |
| --- | --- | --- | --- |
| ***Cytochrome c oxidases*** | | | |
| **cbb_3_-1** | *ccoN1O1Q1P1* | High | Constitutive expression |
| **cbb_3_-2** | *ccoN2O2Q2P2* | High | Low O_2_, stationary phase |
| **aa_3_** | *coxBAC* | Low | Nutrient starvation |
| ***Quinol oxidases*** | | | |
| **bo_3_** | *cyoABCDE* | Low | Iron starvation |
| **CIO** | *cioAB* | Low | Cyanide, copper starvation, inhibition of other oxidases |
| ***Reductase*** | | | |
| **NO reductase** | *norCBD* | Low | Denitrification process, anaerobic respiration |

**Table S2.** Different terminal oxidases of *P. aeruginosa* respiratory chain.

According to (1, 2).

**Table S3:** Impact of DCCP (inhibitor of ATP synthase) on the MIC of aminoglycoside and β-lactam antibiotics.

| **Strains** | **MICs of antibiotics (µg/mL) +/- DCCD** | | | | | | | |
| --- | --- | --- | --- | --- | --- | --- | --- | --- |
|  | **Gentamicin** | | **Tobramycin** | | **Aztreonam** | | **Ticarcillin** | |
| ***+ DCCD (µg/mL)*** | ***0*** | ***50*** | ***0*** | ***50*** | ***0*** | ***50*** | ***0*** | ***50*** |
| **PA14** | 0.5 | 0.25 | 0.25 | 0.125 | 4 | 8 | 32 | 64 |
| **A1** | 0.125 | 0.03 | 0.06 | 0.03 | 32 | 32 | 256 | 256 |
| **A2** | 0.5 | 0.25 | 0.25 | 0.125 | 16 | 16 | 128 | 128 |
| **A3** | 0.125 | 0.06 | 0.06 | 0.06 | 8 | 8 | 64 | 128 |

DCCD (N,N’-dicyclohexylcarbodiimide) is a classical inhibitor of the FOF1-ATP synthase.

**Table S4**. Impact of squalamine on the MIC of aminoglycoside antibiotics.

| **Strains** |  | **MICs of antibiotics (µg/mL)** | | | | | | |
| --- | --- | --- | --- | --- | --- | --- | --- | --- |
|  | **Gentamicin** | | | |  | **Tobramycin** | | |
| ***+ Squalamine (µg/mL)**** | ***0*** | | ***2*** | ***4*** |  | ***0*** | ***2*** | ***4*** |
| **PA14** | 0.5 | | 0.5 | 0.5 |  | 0.25 | 0.25 | 0.25 |
| **A1** | 0.125 | | 0.125 | 0.06 |  | 0.06 | 0.06 | 0.03 |
| **A2** | 0.5 | | 0.5 | 0.5 |  | 0.25 | 0.25 | 0.25 |
| **A3** | 0.125 | | 0.06 | 0.03 |  | 0.06 | 0.03 | 0.015 |

*Squalamine is a natural aminosterol disrupting the bacterial membrane.

| **Table S5.** Mutations in the *atp* cluster found in clinical strains of *P. aeruginosa* (among the 2,500 genomic sequence available at the *Centre National de Reference de la resistance aux antibiotiques,* Besançon, France).   \| **DNA region** \| **Number of strains mutated**  **(*n* = 282)** \| **With deleterious mutations (*n* = 94) *^b^*** \| \| \| --- \| --- \| --- \| --- \| \| **Number of isolates** \| **Substitution** \| \| **Promoter *^a^*** \| 48 \| Unknown (5) *^c^* \| / \| \| ***atpI*** \| 84 \| 15 \| Y35F \| \| 1 \| Y35N \| \| 3 \| S59N \| \| 2 \| A83S \| \| 1 \| G103D \| \| ***atpB*** \| 22 \| 15 \| E87K \| \| 1 \| H98Q \| \| 3 \| G99R \| \| 2 \| V113A* \| \| 1 \| G177D \| \| 15 \| F179L \| \| 1 \| E286K \| \| ***atpE*** \| 1 \| No deleterious mutation \| \| \| ***atpF*** \| 81 \| 2 \| A107V \| \| 1 \| E155A \| \| ***atpH*** \| 4 \| 1 \| R9C \| \| 1 \| Q122R \| \| 1 \| A127T* \| \| ***atpA*** \| 17 \| 1 \| Y80C \| \| 2 \| R162C \| \| 1 \| P282S \| \| 1 \| Q409R \| \| 1 \| A503V* \| \| ***atpG*** \| 11 \| 3 \| I11V \| \| 1 \| N173S \| \| 1 \| K174T \| \| 1 \| L215F \| \| 2 \| L262P \| \| ***atpD*** \| 8 \| 1 \| M55T \| \| 1 \| G98S \| \| 1 \| Y205C \| \| 2 \| A219T \| \| 1 \| A356V \| \| ***atpC*** \| 6 \| 2 \| H6Y \| \| 1 \| G19S \|   *^a^* : Promoter region correspond to the 120-bp upstream from the ATG of gene *atpI.*  *^b^* : according to PolyPhen-2, score: >0.6 is deleterious, between 0.4 and 0.6 is probably deleterious (*).  ^c^ : Impact of mutations in this region is unknown, with the exception of 5 isolates with the same -10/-35 mutated region as the A1 mutant. |
| --- | --- | --- | --- | --- | --- | --- | --- | --- | --- | --- | --- | --- | --- | --- | --- | --- | --- | --- | --- | --- | --- | --- | --- | --- | --- | --- | --- | --- | --- | --- | --- | --- | --- | --- | --- | --- | --- | --- | --- | --- | --- | --- | --- | --- | --- | --- | --- | --- | --- | --- | --- | --- | --- | --- | --- | --- | --- | --- | --- | --- | --- | --- | --- | --- | --- | --- | --- | --- | --- | --- | --- | --- | --- | --- | --- | --- | --- | --- | --- | --- | --- | --- | --- | --- | --- | --- | --- | --- | --- | --- | --- | --- | --- | --- | --- | --- | --- | --- |

| **Table S6.** Expression of genes of the *atp* operon, in the reference strain PA14, 15 minutes after exposure to 350 µg/mL CNA (transcriptomic analysis, laboratory data) compared with that of genes coding for other plasmic membrane proteins.   \| ***PA number*** \| ***Gene*** \| ***Function*** \| ***Expression level*** \| \| --- \| --- \| --- \| --- \| \| ***Genes of the atp operon*** \| \| \| \| \| *PA5558* \| *atpF* \| *F0F1 ATP synthase subunit B* \| ***-3.13*** \| \| *PA5553* \| *atpC* \| *F0F1 ATP synthase subunit epsilon* \| ***-3.38*** \| \| *PA5557* \| *atpH* \| *F0F1 ATP synthase subunit delta* \| ***-3.56*** \| \| *PA5555* \| *atpG* \| *F0F1 ATP synthase subunit gamma* \| ***-3.68*** \| \| *PA5554* \| *atpD* \| *F0F1 ATP synthase subunit beta* \| ***-3.68*** \| \| *PA5556* \| *atpA* \| *F0F1 ATP synthase subunit alpha* \| ***-4.22*** \| \| ***Genes coding for other plasmic membrane proteins*** \| \| \| \| \| *PA2648* \| *nuoM* \| *NADH dehydrogenase subunit M* \| *-1.72* \| \| *PA4418* \| *ftsI* \| *penicillin-binding protein 3* \| *-1.70* \| \| *PA3489* \|  \| *Na^+^-translocating NADH-quinone reductase subunit E* \| *-1.67* \| \| *PA2953* \|  \| *ubiquinone oxidoreductase* \| *-1.32* \| \| *PA2640* \| *nuoE* \| *NADH dehydrogenase subunit E* \| *-1.27* \| \| *PA5045* \| *ponA* \| *penicillin-binding protein 1A* \| *-1.22* \| \| *PA3887* \| *nhaP* \| *Na+/H+ antiporter NhaP* \| *-1.18* \| \| *PA2951* \| *etfA* \| *electron transfer flavoprotein subunit alpha* \| *-1.16* \| \| *PA4538* \| *ndh* \| *NADH dehydrogenase* \| *-1.14* \| \| *PA2580* \|  \| *NADPH specific quinone oxidoreductase* \| *-1.06* \| \| *PA1556* \| *ccoO2* \| *cbb3-type cytochrome c oxidase subunit II* \| *-1.05* \| \| *PA1555* \| *ccoP2* \| *cytochrome c oxydase, cbb3-type subunit* \| *-1.04* \| \| *PA0509* \| *nirN* \| *c-type cytochrome* \| *-1.04* \| \| *PA2638* \| *nuoB* \| *NADH dehydrogenase subunit B* \| *-1.01* \| \| *PA0524* \| *norB* \| *nitric-oxide reductase subunit B* \| *1.03* \| \| *PA1582* \| *sdhD* \| *succinate dehydrogenase (D subunit)* \| *1.09* \| \| *PA2272* \| *pbpC* \| *penicillin-binding protein 3A* \| *1.13* \| \| *PA1082* \| *flgG* \| *flagellar basal body rod protein FlgG* \| *1.13* \| \| *PA5063* \| *ubiE* \| *ubi/menaquinone biosynthesis methyltransferase* \| *1.20* \| |
| --- | --- | --- | --- | --- | --- | --- | --- | --- | --- | --- | --- | --- | --- | --- | --- | --- | --- | --- | --- | --- | --- | --- | --- | --- | --- | --- | --- | --- | --- | --- | --- | --- | --- | --- | --- | --- | --- | --- | --- | --- | --- | --- | --- | --- | --- | --- | --- | --- | --- | --- | --- | --- | --- | --- | --- | --- | --- | --- | --- | --- | --- | --- | --- | --- | --- | --- | --- | --- | --- | --- | --- | --- | --- | --- | --- | --- | --- | --- | --- | --- | --- | --- | --- | --- | --- | --- | --- | --- | --- | --- | --- | --- | --- | --- | --- | --- | --- | --- | --- | --- | --- | --- | --- | --- | --- | --- | --- | --- | --- | --- | --- | --- |

**Table S7.** Strains and plasmids used in the study.

| **Stains** | **Description** | **References** |  |
| --- | --- | --- | --- |
| ***Pseudomonas aeruginosa*** | | | |
| PA14 | Wild type reference strain, susceptible to antibiotics | (3) |  |
| PA14ΔXY | PA14-derived *mexXY* deleted mutant | This study |  |
| PA14*atpI*::MrT7 | Mutant 45399 from the PA14 transposon insertion mutant library inactivated in *atpI* with the MrT7 transposon | (3) |  |
| A1 | PA14-derived mutant resistant to CNA due to a -A_486_ deletion in *nalC* and harbouring a -C deletion in the promoter of *atpI* | (4) |  |
| A2 | PA14-derived mutant resistant to CNA due to a T_24_P substitution in NalC | (4) |  |
| A3 | PA14-derived mutant resistant to CNA harbouring a P_305_S substitution in AtpD | (4) |  |
| A1∆XY | CNA-A1-derived *mexXY* deleted mutant | This study |  |
| A3∆XY | CNA-A3-derived *mexXY* deleted mutant | This study |  |
|  |  |  |  |
| ***Escherichia coli*** | | | |
| CC118*λpir* | CC118 lysogenic for phage *λpir* | (5) |  |
| HB101 | *subE44 subF58 hsdS3*(r_B_^-^ m_B_^-^) *recA13 ara-14 proA2 lacY1 galK2 rpsL20 xyl-5 mtl-1* | (6) |  |
| HB101(pRK2013) | Strain HB101 containing helper plasmid pRK2013 for conjugation (*mob1, tra1, colE1,* Kan^R^) | (7) |  |
| CC118*λpir*(pKNG101) | Strain CC118*λpir* containing suicide vector pKNG101 (*oriR6K, sacB,* Str^R^) | (8) |  |
|  |  |  |  |

Kan^R^, Gen^R^, Str^R^: selective plasmid markers conferring resistance to kanamycin, gentamicin and streptomycin, respectively.

**Table S8.** Primers used for gene inactivation, site-directed mutagenesis, RT-qPCR and sequencing experiments.

| **Primer** | **Sequence (5’→ 3’)** | **Tm (°C)** | **Source** |
| --- | --- | --- | --- |
| **Genes inactivation** (complementary regions are in red and green colours) | | | |
| *mexXY* | |  |  |
| iPA14*mexXY*-1 | cccccccctgcaggtcgacggcgttcgcacttgaggtag | 86.2 | This study |
| iPA14*mexXY*-2 | tgctccgtcggtgtccctcgattcgtgaac | 76.2 |  |
| iPA14*mexXY*-3 | cgagggacaccgacggagcaagcctgatg | 77.7 |  |
| iPA14*mexXY*-4 | ttctacttatggtacccgggctacgacaaggccagcgatc | 81 |  |
| **RT-qPCR** | | | |
| *mexB1* | ATCCGCCAGACCATCGCCA | 60 | (9) |
| *mexB2* | CATCACCAGGAACACGAGGAGG | 60 |  |
| *mexY1A* | TTACCTCCTCCAGCGGC | 60 | (10) |
| *mexY1B* | GTGAGGCGCGCGTTGTG | 60 |  |
| *atpI* | AGATTGCTGCTCGTCCAACT | 58.4 | This study |
| *atpI* | CCCAGCAGTCCGGAATATCC | 62.5 |  |
| *atpB* | TTCGGGTTACATCCAGCACC | 60.5 | This study |
| *atpB* | CATTTCCTTGGCTTGCTCGG | 60.5 |  |
| *atpE* | TTGCTCTGCTGATTGGCCTG | 60.5 | This study |
| *atpE* | TTTCACCTGCAGCATCGGAA | 58.4 |  |
| *atpF* | TCATGAGAAAGCGGGCCAG | 60.5 | This study |
| *atpF* | CTGGTTAGCGCGTTTCTTCG | 60.5 |  |
| *atpH* | AGAGCCTGATCGATGTGTGC | 60.5 | This study |
| *atpH* | CTCGACCGACTTTTCCTGCT | 60.5 |  |
| *atpA* | CACGAAACGAAGGCACCATC | 60.5 | This study |
| *atpA* | TTCAGCGCCATACCGTAGAC | 60.5 |  |
| *atpG* | TCATGGTCGAGCGTGAAGTC | 60.5 | This study |
| *atpG* | TAGCCGCTCATGTCCTTGAC | 60.5 |  |
| *atpD* | CGGATCATGGACGTACTGGG | 62.5 | This study |
| *atpD* | TCACCTTGATGCCGGTTTCC | 60.5 |  |
| *atpC* | GGCCTGGTCGAGATGGTTAT | 60.5 | This study |
| *atpC* | GTAGTACACCTCCTGCTCGC | 62.5 |  |
| *rpsL3* | GCAACTATCAACCAGCTGGTG | 60 | (11) |
| *rpsl5* | GCTGTGCTCTTGCAGGTTGTG | 60 |  |

The complementary sequences required for overlapping PCR and plasmid integration are shown in colour.

**Figure S1.** Map of the cluster *atpIBEFHAGDC* encoding the subunits of the ATP synthase of *P. aeruginosa* (*atpI,* ATP synthase protein I; *atpB*, ATP synthase A chain; *atpE*, ATP synthase C chain, *atpF*, ATP synthase B chain; *atpH*, ATP synthase delta chain; *atpA*, ATP synthase alpha chain; *atpG*, ATP synthase gamma chain; *atpD*, ATP synthase beta chain; *atpC*, ATP synthase epsilon chain)*.* **(A)** In the box, is indicated the sequence upstream of the gene *atpI* containing the promoter of *atpI* (according to SoftBerry.com). The A1 mutant has a deletion of one C in the promoter region (in red) shortening (from 14 to 13 bases) the space between the -10 and -35 regions. **(B)** The structure of the bacterial ATP synthase with corresponding alpha, beta, delta, gamma and epsilon chains has been borrowed from (12).

*atpI*

*atpC*

*atpD*

*atpG*

*atpA*

*atpB*

*atpE*

*atpF*

*atpH*


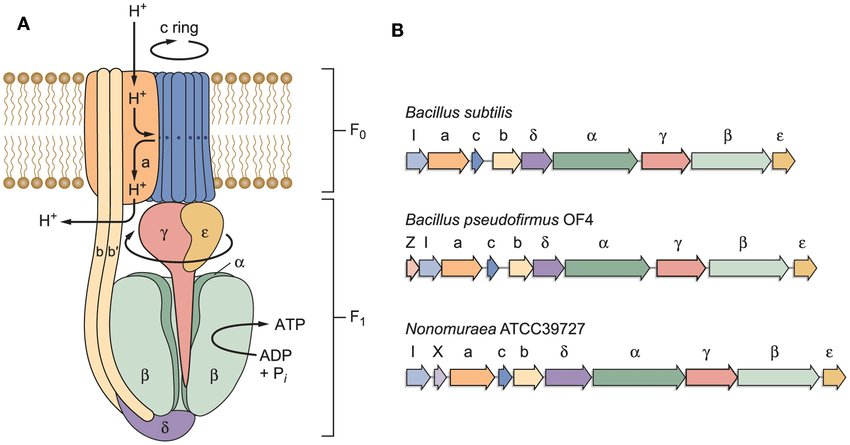


**B**

1000-bp

**A**

-35 -10

**TTGAAC**AGGGGGGACTCCCC**CCCTATACT**CTGCGCGCTATTTTGTGGGCACTTTTTATGCCAAGTCGTTGATTTGGCAGTGCCGACACCAGAAGACTGAGAGCGCGATGGAGTCCCGC**ATG**

**Figure S2**. Antibiotics susceptibility profile of the transposition mutant PA14*atpI*::MrT7 inactivated in *atpI* compared with PA14*.* The inactivation of *atpI* by the transposon MrT7 (PA14*atpI*::MrT7 resistant to gentamicin **GME**, *right*) induces slight hypersusceptibility to aminoglycosides (tobramycin **TMN, and** amikacin **AKN,** in yellow) and reduced susceptiblity to β-lactams antibiotics (piperacillin-tazobactam **PTZ**, cefotaxime **CTX**, ticarcillin **TIC**, and ticarcillin-clavulanic acid **TCC,** in pink) compared with the parental strain PA14-Washington (*left*) (gift from Romé Vouhloux and Geveniève Ball, Laboratoire de Chimie Bactérienne, Institut de Microbiologie de la Méditerranée, Marseille - France).

**PA14-Washington PA14*atpI*::MrT7**


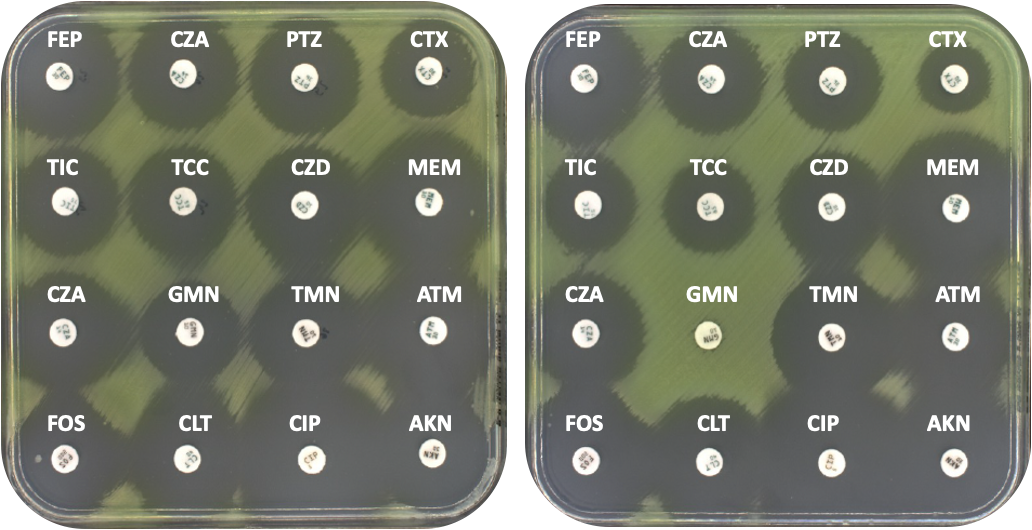


**Figure S3.** Measurement of ATP production in A1, A2 and A3 mutants by luminescence (RLU). Total ATP production was obtained after measurement by luminescence of luciferin oxidation by luciferase in lysed cells, at the beginning (A_600nm_= 0.3) of the exponential phase. Significance was determined by one-way ANOVA followed by Dunnett’s test (*: *p* value < 0.05; **: *p* value <0.01).

**

*

*

**Figure S4.** Elements of *P. aeruginosa* respiratory chain. The ATP synthase activity and the membrane potential (∆Ψ) depends on that of the respiratory chain which is fuelled at two levels: (i) by NADH dehydrogenase (Complex I), a membrane-bound oxidoreductase which catalyses the reduction of ubiquinone (Q) to ubiquinol (QH_2_), producing NAD^+^, two protons (2H^+^) and two electrons (2e^-^); (ii) by succinate dehydrogenase (Complex II), which captures electrons from succinate, converting it to fumarate, also producing 2H^+^ and 2e^-^. Electrons are then transferred to a super complex constituted by Complexes III and IV, respectively a ubiquinone oxidase and a terminal oxidase, which ultimately reduces oxygen to water. Several terminal oxidases exist in Gram-negative bacteria (Cbb3_1_, Cbb3_2_, Aa3, Bo3, Cio, and NorB), each responding to different environmental conditions. The genes encoding respiratory chain components are indicated below the figure; those tested by RT-qPCR are shown in red. Adapted from (1, 13) (Biorender). **(A)** Reference strain PA14, **(B)** hypersusceptible mutants A1 and A3 affected in ATP synthase.

**A**

*
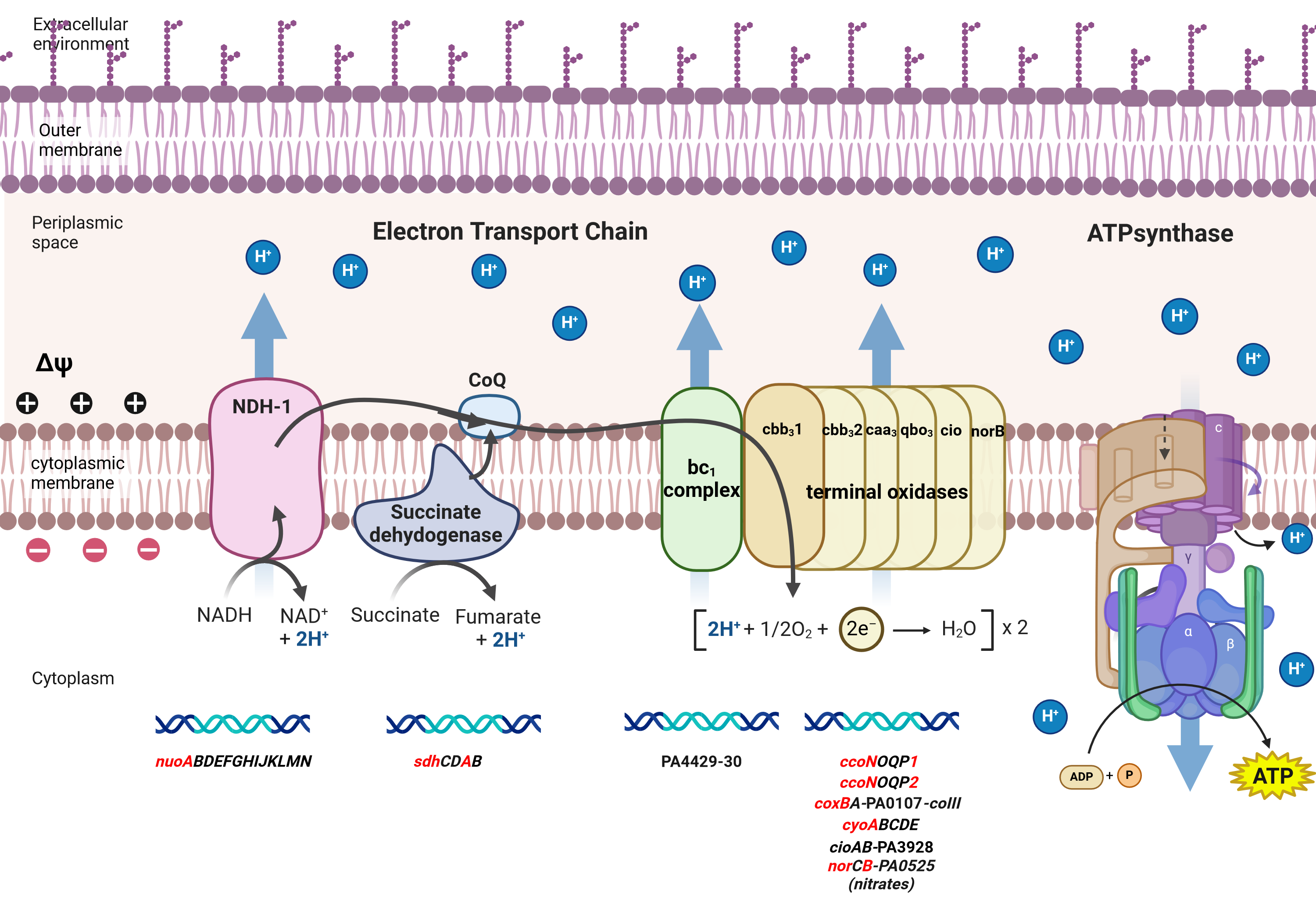
*

**B**


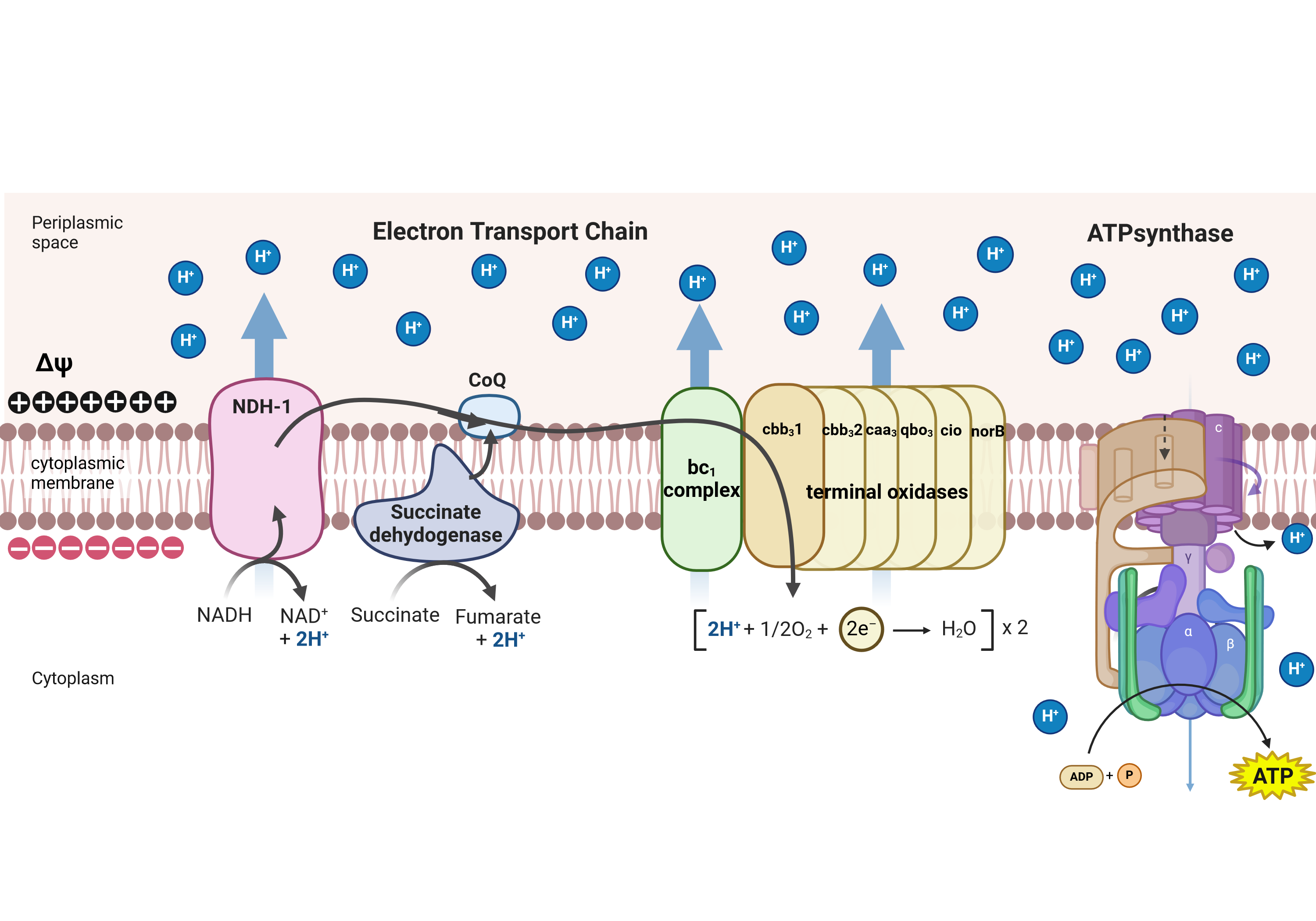


**Figure S5.** Measurement of the Proton Motive Force (PMF) using fluorescence. The PMF is assessed by employing the fluorescent molecular marker BCECF/AM, which shows increased fluorescence as cytoplasmic pH rises (correlated with a decrease in periplasmic pH). When DCCD, an inhibitor of ATP synthase, is applied (50 µg/mL), protons accumulate in the periplasmic space, leading to an increase in cytoplasmic pH, which in turn increases the intensity of BCECF fluorescence. An ANOVA test was followed by a Tukey HSD post hoc test to identify differences in fluorescence; bars marked with different letters indicate statistically significant differences (p_value <0.01).


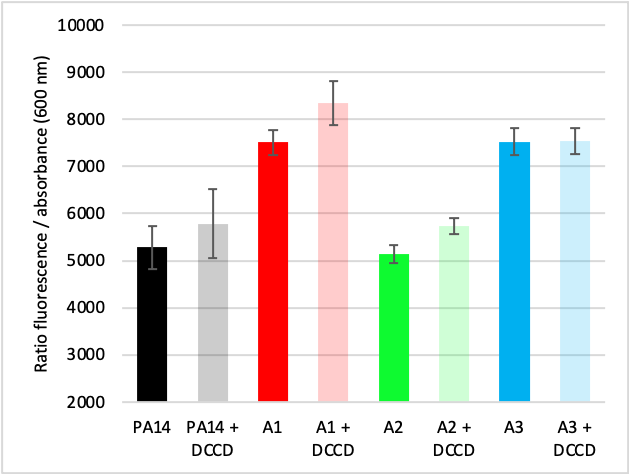


a

a

a

a

b

b

b

b

**References**

1. Arai H. 2011. Regulation and Function of Versatile Aerobic and Anaerobic Respiratory Metabolism in *Pseudomonas aeruginosa*. Front Microbiol 2:103.

2. Arai H, Kawakami T, Osamura T, Hirai T, Sakai Y, Ishii M. 2014. Enzymatic characterization and in vivo function of five terminal oxidases in *Pseudomonas aeruginosa*. J Bacteriol 196:4206-15.

3. Liberati NT, Urbach JM, Miyata S, Lee DG, Drenkard E, Wu G, Villanueva J, Wei T, Ausubel FM. 2006. An ordered, nonredundant library of *Pseudomonas aeruginosa* strain PA14 transposon insertion mutants. Proc Natl Acad Sci U S A 103:2833-8.

4. Tetard A, Gaillot S, Dubois E, Aarras S, Valot B, Phan G, Plesiat P, Llanes C. 2022. Exposure of *Pseudomonas aeruginosa* to cinnamaldehyde selects multidrug resistant mutants. Antibiotics (Basel) 11:1790.

5. Herrero M, de Lorenzo V, Timmis KN. 1990. Transposon vectors containing non-antibiotic resistance selection markers for cloning and stable chromosomal insertion of foreign genes in Gram-negative bacteria. J Bacteriol 172:6557-67.

6. Lacks S, Greenberg B. 1977. Complementary specificity of restriction endonucleases of *Diplococcus pneumoniae* with respect to DNA methylation. J Mol Biol 114:153-68.

7. Ditta G, Stanfield S, Corbin D, Helinski DR. 1980. Broad host range DNA cloning system for gram-negative bacteria: construction of a gene bank of *Rhizobium meliloti*. Proc Natl Acad Sci U S A 77:7347-51.

8. Kaniga K, Delor I, Cornelis GR. 1991. A wide-host-range suicide vector for improving reverse genetics in Gram-negative bacteria: inactivation of the *blaA* gene of *Yersinia enterocolitica*. Gene 109:137-41.

9. Hocquet D, Nordmann P, El Garch F, Cabanne L, Plésiat P. 2006. Involvement of the MexXY-OprM efflux system in emergence of cefepime resistance in clinical strains of *Pseudomonas aeruginosa*. Antimicrob Agents Chemother 50:1347-51.

10. Jeannot K, Sobel ML, El Garch F, Poole K, Plésiat P. 2005. Induction of the MexXY efflux pump in *Pseudomonas aeruginosa* is dependent on drug-ribosome interaction. J Bacteriol 187:5341-6.

11. Dumas JL, van Delden C, Perron K, Köhler T. 2006. Analysis of antibiotic resistance gene expression in *Pseudomonas aeruginosa* by quantitative real-time-PCR. FEMS Microbiol Lett 254:217-25.

12. Preiss L, Hicks DB, Suzuki S, Meier T, Krulwich TA. 2015. Alkaliphilic bacteria with impact on industrial applications, concepts of early life forms, and bioenergetics of ATP synthesis. Front Bioeng Biotechnol 3:75.

13. Di Trani JM, Gheorghita AA, Turner M, Brzezinski P, Adelroth P, Vahidi S, Howell PL, Rubinstein JL. 2023. Structure of the bc(1)-cbb(3) respiratory supercomplex from *Pseudomonas aeruginosa*. Proc Natl Acad Sci U S A 120:e2307093120.
